# Cellular prion protein distribution in the vomeronasal organ, parotid, and scent glands of white-tailed deer and mule deer

**DOI:** 10.1080/19336896.2022.2079888

**Published:** 2022-05-29

**Authors:** Anthony Ness, Aradhana Jacob, Kelsey Saboraki, Alicia Otero, Danielle Gushue, Diana Martinez Moreno, Melanie de Peña, Xinli Tang, Judd Aiken, Susan Lingle, Debbie McKenzie

**Affiliations:** a Department of Biological Sciences, University of Alberta, Edmonton, Alberta, Canada; b Centre for Prions and Protein Folding Diseases, Edmonton, Alberta, Canada; c Department of Biology, University of Winnipeg, Winnipeg, Manitoba, Canada; d Enfermedades Transmisibles Emergentes, Universidad de ZaragozaCentro de Encefalopatías y , Zaragoza, Spain; e Department of Biochemistry, University of Alberta, Edmonton, Alberta, Canada; f Department of Agriculture, Food and Nutritional Sciences, University of Alberta, Edmonton, Alberta, Canada

**Keywords:** Prion, chronic wasting disease, sex differences, species differences, disease prevalence, cervid, protein expression, glands

## Abstract

Chronic wasting disease (CWD) is a contagious and fatal transmissible spongiform encephalopathy affecting species of the cervidae family. CWD has an expanding geographic range and complex, poorly understood transmission mechanics. CWD is disproportionately prevalent in wild male mule deer and male white-tailed deer. Sex and species influences on CWD prevalence have been hypothesized to be related to animal behaviours that involve deer facial and body exocrine glands. Understanding CWD transmission potential requires a foundational knowledge of the cellular prion protein (PrP^C^) in glands associated with cervid behaviours. In this study, we characterized the presence and distribution of PrP^C^ in six integumentary and two non-integumentary tissues of hunter-harvested mule deer (*Odocoileus hemionus*) and white-tailed deer (*O. virginianus*). We report that white-tailed deer expressed significantly more PrP^C^ than their mule deer in the parotid, metatarsal, and interdigital glands. Females expressed more PrP^C^ than males in the forehead and preorbital glands. The distribution of PrP^C^ within the integumentary exocrine glands of the face and legs were localized to glandular cells, hair follicles, epidermis, and immune cell infiltrates. All tissues examined expressed sufficient quantities of PrP^C^ to serve as possible sites of prion initial infection, propagation, and shedding.

## Introduction:

Chronic wasting disease (CWD) is a contagious prion disease of free-ranging and captive species of the Cervidae family including mule deer (*Odocoileus hemionus*), white-tailed deer (*Odocoileus virginianus*), Rocky Mountain elk (*Cervus elaphus nelsoni*), reindeer/caribou (*Rangifer tarandus*), and moose (*Alces alces*). CWD, like all prion diseases, is characterized by a long preclinical period followed by a rapid clinical onset and decline leading to death. In a CWD-infected individual infectious prions convert healthy cellular prion protein (PrP^C^) into the CWD-associated isoform (PrP^CWD^) through a protein template misfolding mechanism [1,2]. Throughout the course of disease, prions propagate in infected cervids and are shed via body fluids and excreta including saliva, faeces, and urine [3–7]. Although cervids can acquire infectious prions through direct or indirect contact [4,8], the specific mechanisms underlying the transmission of CWD prions in wild and captive populations remains poorly understood. The most accepted route of indirect CWD exposure is the oral-nasal uptake of PrP^CWD^ from environmental fomites including soil particles, vegetation, and salt licks [8–13]. Oral-nasal CWD transmission mechanisms are, however, inadequate for explaining remarkably disproportionate disease prevalence patterns in wild cervid populations whereby males and mule deer are more likely to be infected by CWD relative to females and white-tailed deer [14–19].

Glandular tissues offer an unorthodox, alternative route of CWD transmission that has not been characterized. Cervids have numerous integumentary scent glands on their face and legs [20–23]. Cervid exocrine gland secretory tubules and acini are possible sources of PrP^CWD^ shedding and possible entry points for PrP^CWD^ during direct or indirect pathogen exposure. Integumentary glands are well innervated by the autonomic nervous system including the sympathetic nervous system. These innervations offer possible sites of neuroinvasion by PrP^CWD^. Similarly, integumentary exocrine glands are points of entry for many pathogens [23–26]. Secondary to neuroinvasion at the glands, immune cells may traffic PrP^CWD^ from glands to secondary lymphatic structures for later neuroinvasion. PrP^CWD^ in salivary glands of CWD infected deer [6,27], and high levels of PrP^C^ in mammary glands of healthy animals [28] suggest that PrP^C^ and the capacity for conversion into PrP^CWD^ will be high in other integumentary exocrine glands. The parotid gland (and its associated lymph nodes) has been widely investigated for CWD involvement [6,11,29–34]. The immediate proximity to the oral cavity makes the parotid gland and its paraglandular lymph nodes [35] likely to contact orally ingested PrP^CWD^ and the gland is an intuitive source of CWD infectivity in saliva. Other peripheral cervid glands have been largely neglected in CWD distribution studies. The only reported investigation into integumentary gland PrP^CWD^ presence of the leg or face was by Spraker *et al*., who observed no PrP^CWD^ by immunohistochemistry in the tarsal glands of free-ranging and captive mule deer with end-stage, naturally acquired CWD [31]. A broad range of common deer behaviours, including grooming, sparring, courtship, and dominance interactions bring animals in direct contact with exocrine glands or indirect contact with glandular secretions deposited in the environment [36]. These behaviours also involve contact with known sources of infectious prions (e.g., urine and saliva) and are hypothesized to contribute to CWD transmission and to the increased prevalence of CWD in mule deer and in males of wild populations [16,37–39]. If exocrine glands play a role in CWD infection, our understanding of the body sites that serve as routes of infection, and the behavioural contexts in which CWD transmission occurs, may change dramatically.

Scent-marking is one form of indirect contact that may contribute to the sex bias in CWD prevalence [16,37,40]. Male white-tailed deer interact with scrapes – scent signposts made by bucks involving pawing the ground and interacting with nearby vegetating – more frequently than does [40], and male mule deer and white-tailed deer, but not females, engage in high levels of advertisement activities including nasal-oral marking, antler thrashing, and scrapes [39] support scent marking as a contributor to sex biased CWD prevalence. Kinsell (2010) further proposed a risk model of CWD prion shedding and exposure via the preorbital, forehead, tarsal, and interdigital glands associated with deer marking and scraping behaviours. As yet, biochemical characterization to support CWD involvement with exocrine glands is lacking. In this study, we describe the presence and distribution of PrP^C^ within integumentary exocrine glands of mule deer and white-tailed deer to assess the potential for PrP^CWD^ uptake or shedding from these glands and to determine whether sex and species differences in PrP^C^ in these glands correspond to the sex and species skew in CWD prevalence.

## Methods:

### Tissue collection:

Mule deer and white-tailed deer were harvested by hunters on the Canadian Forces Base (CFB) Wainwright in southeastern Alberta, Canada, between 30 November 2017 and 13 December 2017. Samples from 40 deer with two mule deer that tested positive for CWD being excluded from analysis. For the PrP^C^ survey described we used samples from 15 mule deer (8 males, 7 females), and 16 white-tailed deer (8 of each sex). All animals were ≥1.5 years of age, with a similar distribution in body and antler size for males of the two species. More accurate age estimation by cementum annuli analysis was not available. Glandular tissues were extracted at the time the deer were brought to the hunter-check station, 1.3 to 10.1 hours after the animal was killed. Samples used for biochemical analysis were preferentially selected for in favour of animals with shorter periods of time between animal death and sampling. Ambient temperature during tissue collection ranged from −10°C to 4°C. Samples were frozen for later biochemical analysis or formalin fixed for histology.

**Figure 1.**
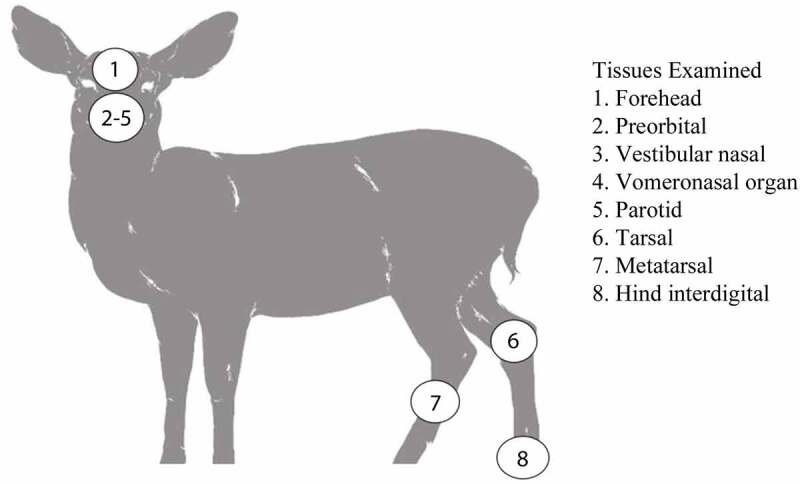
Scent glands and non-integumentary tissues sampled from mule deer and white-tailed deer from Canadian forces base wainwright, Alberta.

The non-integumentary glandular tissue collected included the parotid gland and the anterior portion of the vomeronasal organ. The integumentary glands collected included the facial forehead, preorbital, lateral vestibular nasal glands, and the leg tarsal, metatarsal, and hind interdigital glands (Figure 1).

### Tissue homogenization and protein content determination:

Extracted glandular tissues were trimmed of non-glandular tissue, fat, hair, and secretory exudate. Glandular tissue was excised, minced, weighed, and then homogenized in RIPA lysis buffer (50 mM Tris, 150 mM NaCl, 1% IGEPAL CA-630, 0.25% deoxycholate, 1 mM EDTA, pH 7.4) supplemented with cOmplete™ EDTA-Free Protease Inhibitor Cocktail (Roche Diagnostics GmbH, Switzerland). Samples were mechanically homogenized in a Bead Ruptor 24 (Omni International, USA) bead mill homogenizer in the presence of a cold airflow supplied by an OMNI BR-Cryo cooling unit to minimize heat-denaturation. Glandular tissue was subjected to 25 minutes of high-energy ceramic bead milling-assisted agitation cycles of 10 second intervals of milling followed by 15 seconds of cooling. Homogenate supernatants were collected following a brief microcentrifugation to yield clarified 10% (w/v) gland homogenates for biochemical analysis. Total protein content of clarified 10% (w/v) gland homogenates was determined using a Pierce™ BCA Protein Assay Kit (Thermo Fisher Scientific, USA). Homogenate protein concentrations of deer species and sex were compared by non-repeated-measures two-way ANOVA with GraphPad Prism software (GraphPad Software, USA).

### Western blot analysis:

Determination of total prion protein abundance by western blot under denaturing conditions required atypical conditions to prevent aberrant protein separation. Samples were denatured for 15 minutes at 100°C having first been diluted into one part 10% clarified gland homogenate, one part water, and two parts 5x Laemmeli buffer. Samples (7.5 µL per well) were loaded into 15-well Invitrogen NuPAGE™ 12% Bis-Tris protein gels (Thermo Fisher Scientific, USA). Gels were run slowly at 70 V for 5 hours – the entire gel length – in MOPS (3-(N-Morpholino) propane sulphonic acid) buffer. Proteins were transferred onto Immobilon®-FL (MilliporeSigma, USA) PVDF (polyvinylidene fluoride) membranes at 35 V for 1.5 hours followed by blocking with 2% (w/v) bovine serum albumin in phosphate buffered saline (130 mM NaCl, 7 mM Na_2_HPO_4_ · 7H_2_O, 3 mM NaH_2_PO_4_ · 1H_2_O, pH 7.4). PrP^C^ was probed with the mouse IgG_1_ anti-prion protein SHA31 monoclonal primary antibody (1:10,000) (Cayman Chemical, USA) which binds to an epitope of 148–155 (*Odocoileus* amino acid sequence). Secondary goat anti-mouse IgG (H + L) AP Conjugate (Promega Corporation, USA) detection antibody (1:10,000) was enzymatically developed with AttoPhos® AP Fluorescent Substrate System (Promega Corporation, USA). Membrane fluorescent imaging was performed using an ImageQuant LAS 4000 (GE Life Sciences, USA) system.

Relative protein expression was compared using pixel intensity analysis with ImageJ software. A box enclosing individual western blot lane regions of interest (ROI) ranging from the unglycosylated PrP fragments to the diglycosylated forms of PrP^C^ recognized by the anti-PrP SHA31 antibody (14–37KDa) were used to determine average pixel intensity. Average western blot background-adjusted pixel intensity was compared using Mann-Whitney tests (* p < 0.05, ** p < 0.01, *** p < 0.001).

### Capillary electrophoresis immunoassay:

Unless otherwise stated, all reagents for capillary electrophoresis immunoassays were purchased from Protein Simple (Bio-Techne Corporation, USA). Capillary Western assay analysis was used to compare PrP^C^ in protein concentration-adjusted samples. Clarified 10% (w/v) gland homogenates and 1% white-tailed deer whole-brain homogenate controls were adjusted with capillary assay diluent and master mix to a final protein concentration of 1.5 μg/μL for capillary electrophoresis. PrP^C^ was detected in capillaries with SHA31 primary antibody (1:10,000) and Protein Simple anti-mouse secondary antibody were consistent to that described in Castle, *et al*., 2018 [41]. Samples were loaded into 12–230 kDa 25-capillary cartridges for automated separation and chemiluminescent imaging in a Protein Simple Wes™ machine (Bio-Techne Corporation, USA). Analysis was performed with the associated Compass for Simple Western software. Baseline-fit corrected peak areas (as determined by the perpendicular drop method) corresponding with expected PrP^C^ molecular weights were compared between deer species and sex by two-way ANOVA with additional comparison of sex and species by Bonferroni post-hoc tests for tissues with significant interaction effects.

### Enzyme-linked immunosorbent assay:

The concentration of PrP^C^ in 10% (w/v) clarified gland homogenates was quantified by sandwich enzyme-linked immunosorbent assay (ELISA). Anti-PrP SHA31 (*Odocoileus* sequence epitope of amino acids 148–155) capture antibody (1:5,000) and horseradish peroxidase-conjugated N5 (*Odocoileus* sequence epitope of amino acids 101–104) detection antibodies (1:5,000) were used in custom sandwich ELISA. Recombinant wild-type sequence full-length deer prion protein was used for quantitative standardization (courtesy of Dr. Leonardo Cortez (University of Alberta)). Capture antibody was coated onto high-binding polystyrene strip plates (Greiner Bio-One International, Austria) with 50 mM carbonate-bicarbonate (pH 9.6) buffer. Clarified gland homogenate (25 µL) was diluted into 75 µL of phosphate buffered saline in the ELISA plates, in triplicate. White-tailed deer whole-brain homogenate (1% w/v) was used for comparison. Recombinant deer PrP and brain homogenate controls were diluted to final concentrations of 25% RIPA lysis buffer to match gland samples. Plates were developed with TMB (3,3’,5,5’-tetramethylbenzidine) One Component HRP (horseradish peroxidase) Microwell Substrate (Surmodics, Inc., USA) chromogen and scanned at 650 nm and, following addition of 2 N sulphuric acid stop solution to be scanned at 450 nm. Quantified gland homogenate concentrations of PrP^C^ variants detectable by the SHA31-N5 sandwich combination were compared between deer species and sex by two-way ANOVA with additional comparison of sex and species by Bonferroni post-hoc tests for tissues with significant interaction effects.

### Histology and immunohistochemical detection of the cellular prion protein in gland tissues:

Formalin fixed glands were trimmed and embedded in paraffin. 4 µm thick sections were cut using a microtome and mounted on glass slides. Sections were then stained using the haematoxylin and eosin (H&E) protocol to identify the histological structures of the glands. Immunohistochemical labelling for the detection of cellular prion protein (PrP^C^) was performed similarly to that previously described [42]. Briefly, tissue sections were deparaffinized and dehydrated by immersion in xylene and decreasing concentrations of ethanol (100%, 95% and 70%). Epitope retrieval was performed by hydrated autoclaving at 121°C in citrate buffer (pH 6) for 5 minutes. Endogenous peroxidase was blocked using a 3% hydrogen peroxide solution for 12 minutes. Sections were exposed to 5% goat serum for 1 h to block non-specific sites followed by 15 minutes of blocking with avidin and biotin (Vector Laboratories, USA), respectively. Immunodetection was completed by incubating the samples with the monoclonal antibody BAR224 (1:50; Cayman Chemical, USA) overnight at 4°C followed by 1 h of incubation with Immun-Star anti-mouse HRP secondary antibody (1:250) (Bio-Rad, USA). SHA31 is not suitable for immunohistochemistry. DAB (3,3′-Diaminobenzidine) (MilliporeSigma, USA) was used as the HRP chromogen substrate. After counterstaining with haematoxylin, sections were mounted with DPX (distyrene, plasticizer and xylene) mounting medium (MilliporeSigma, USA). Control slides in which incubation with the primary antibody was omitted were used for specificity controls of the technique. Slides were scanned with a NanoZoomer 2.ORS (Hamamatsu Photonics K.K., Japan) and the images analysed with the manufacturer’s NDP.view2 software. For each tissue examined by immunohistochemistry, six deer of each species were used (three of each sex).

## Results:

### Integumentary gland sample gross examination:

Species- and sex-dependent gross morphological differences of the glands were consistent with historical descriptions. Male forehead gland dermal and epidermal layers were thicker than those of female white-tailed deer [22]. Sampled male mule deer were similarly observed to possess thicker forehead glandular tissue than females. Mule deer metatarsal glands are considerably larger than those of white-tailed deer [21,43,44]. The interdigital glands of white-tailed deer possessed more externalized ceraceous secreta than mule deer – consistent with historical observations [21]. The preorbital gland sacs of mule deer were larger than white-tailed deer [43,44]. We observed more ceraceous accumulations in mule deer preorbital sacs relative to sampled white-tailed deer. The anal and preputial glands were not collected as they were damaged or destroyed during hunter field dressing.

### Clarified gland homogenate total protein concentration:

Total protein concentration of uninfected clarified 10% (w/v) mule deer and white-tailed deer gland homogenates was generally consistent between species and sex for individual glands (Supplementary Figure 1, Supplementary Table 1). Gland homogenate total protein concentration was not significantly influenced by species or sex in 4 of 7 glands – the preorbital, vestibular nasal, tarsal, and metatarsal glands – and the vomeronasal organ. Total protein content was significantly higher in the female forehead and parotid glands relative to males. Species significantly influenced total protein content in parotid and interdigital glands. Protein concentration was higher in white-tailed deer parotid gland homogenates while mule deer interdigital glands yielded higher protein concentrations. The average total protein concentrations of clarified 10% (w/v) homogenates from the integumentary glands and the vomeronasal organ of both deer species ranged from 3.09 µg/µL to 5.86 µg/µL. The parotid gland homogenate protein content was notably higher than other glands examined with an average protein concentration of 9.76 µg/µL in mule deer and 10.85 µg/µL in white-tailed deer. Reference control 10% (w/v) white-tailed deer whole-brain homogenate (not clarified) total protein concentration averaged 9.64 µg/µL.

### Western blot analysis of PrP^C^ expression in exocrine glands:

**Figure 2.**
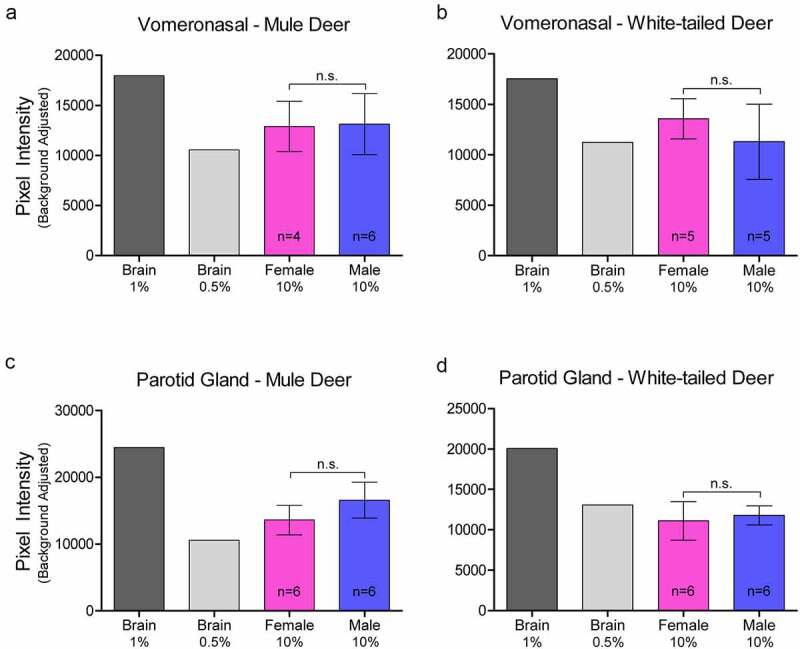
Relative pixel intensity analysis of PrP^C^ expression in non-integumentary cranial exocrine clarified 10% (w/v) gland homogenates of mule deer (a, c) and white-tailed deer (b, d). Background-adjusted average pixel intensities of PrP^C^ bands of the vomeronasal organs (a-b) and parotid glands (c-d) were compared between lanes of SDS-PAGE PVDF membranes probed with anti-PrP Sha31. Unclarified white-tailed deer whole brain homogenate was used for reference. Sample size, mean, 95% confidence intervals, and significance by Mann-Whitney tests are shown.

**Figure 3.**
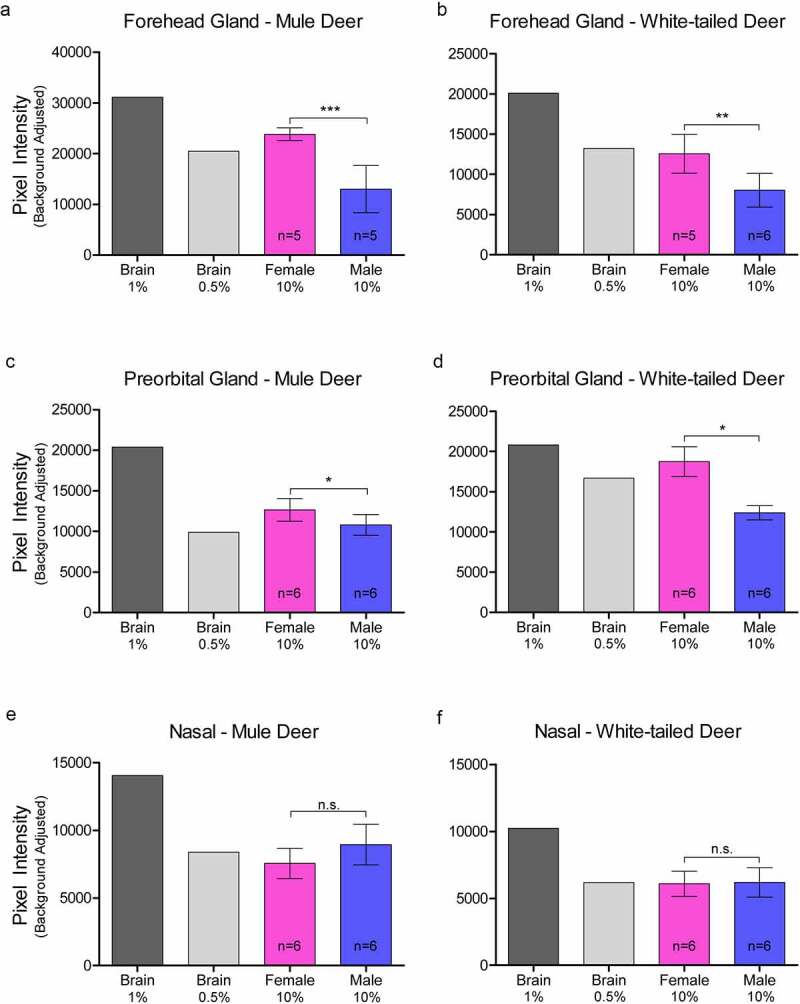
Relative pixel intensity analysis of PrP^C^ expression in facial integumentary clarified 10% (w/v) gland homogenates of mule deer (A, C, E) and white-tailed deer (B, D, F). Background-adjusted average pixel intensities of PrP^C^ bands of forehead (a-b), preorbital (c-d), and vestibular nasal glands were compared between lanes of SDS-PAGE PVDF membranes probed with anti-PrP Sha31. Unclarified white-tailed deer whole brain homogenate was used for reference. Sample size, mean, 95% confidence intervals, and significance by Mann-Whitney tests are shown.

**Figure 4.**
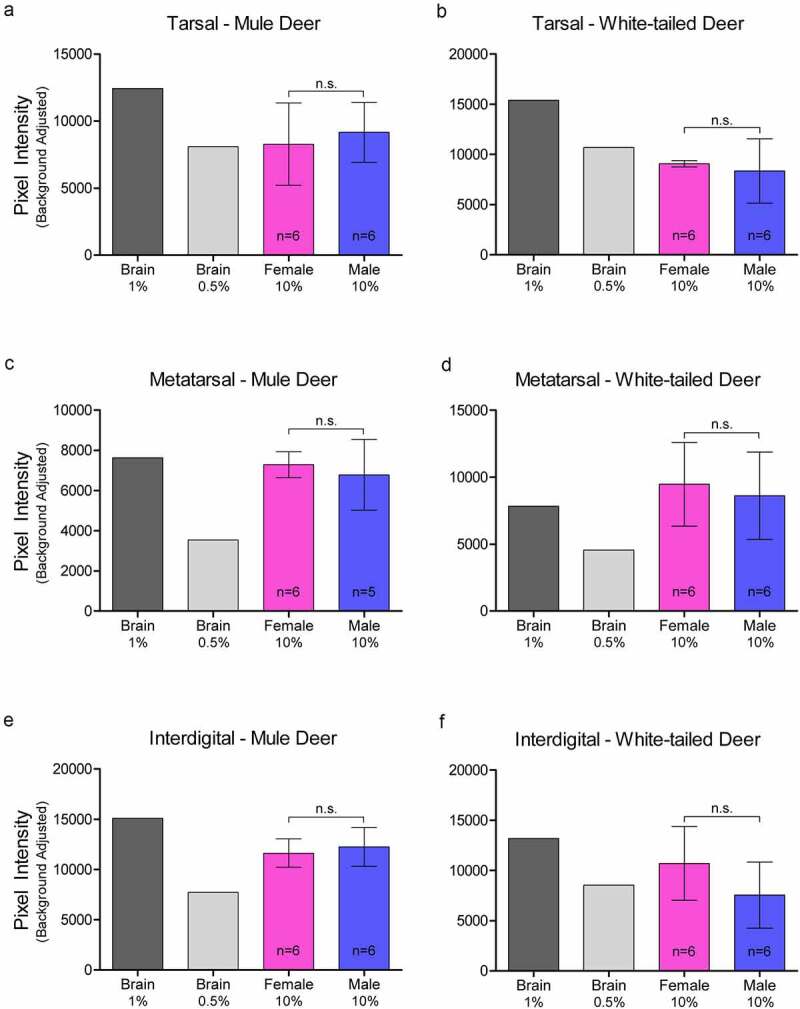
Relative pixel intensity analysis of PrP^C^ expression in leg integumentary clarified 10% (w/v) gland homogenates of mule deer (A, C, D) and white-tailed deer (B, D, F). Background-adjusted average pixel intensities of PrP^C^ bands of tarsal (a-b), metatarsal (c-d), and interdigital (e-f) glands were compared between lanes of SDS-PAGE PVDF membranes probed with anti-PrP Sha31. Unclarified white-tailed deer whole brain homogenate was used for reference. Sample size, mean, 95% confidence intervals, and significance by Mann-Whitney tests are shown.

Relative PrP^C^ protein expression in uninfected deer was detected by western blot in all exocrine glands examined (Supplementary Figure 2). Male and female PrP^C^ expression was compared for each species. 10% (w/v) gland homogenate (not adjusted for total protein content) PrP^C^ detected by western blot with anti-PrP SHA31 antibody was comparable to 0.5–1% white-tailed deer whole-brain homogenates (Figures 2–4). Broadly, facial and leg gland homogenates express at least 10-fold less PrP^C^ than 10% brain homogenates. Glycosylation patterns of PrP^C^ varied between glands and were distinguishably different from brain homogenate-derived PrP^C^ (Supplementary Figure 2). Among a representative panel of deer (4–6 deer of each sex), female deer of both species expressed significantly more PrP^C^ than their male counterparts in the forehead and preorbital integumentary glands as determined by pixel intensity analysis (Figure 3).

### Protein-adjusted PrP^C^ expression examined by capillary electrophoresis immunoassay:

**Figure 5.**
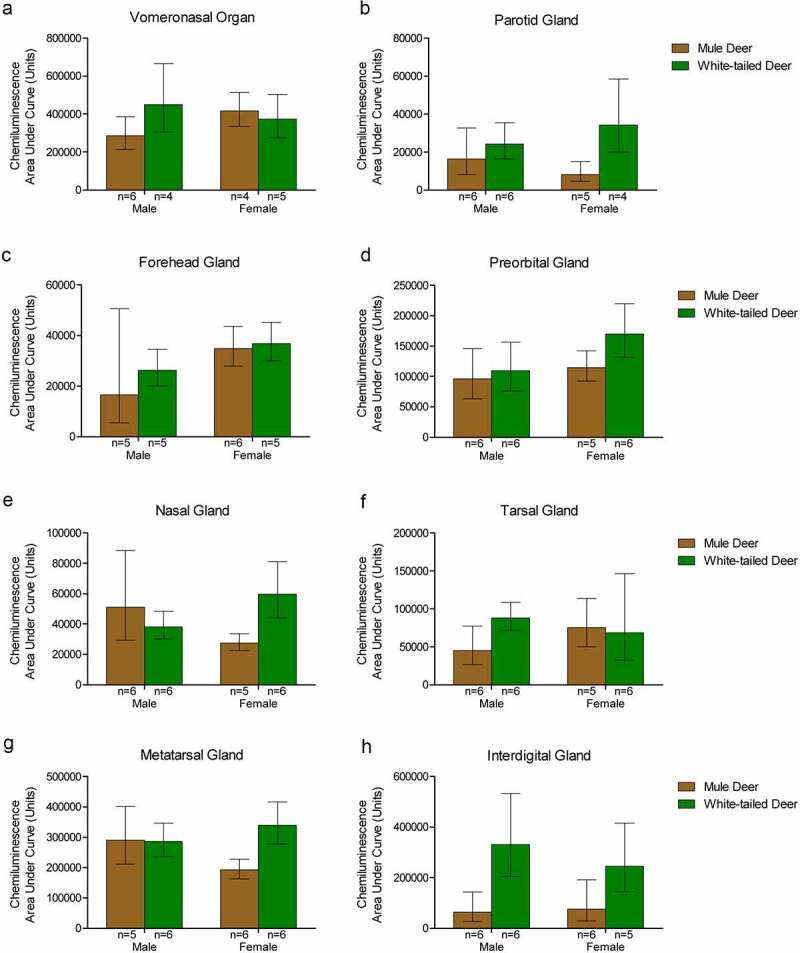
Protein concentration-adjusted PrP^C^ protein expression in deer exocrine glands. Capillary electrophoresis immunoassay chemiluminescence sample size, mean, and 95% confidence intervals of clarified 10% (w/v) deer gland homogenates prepared in RIPA buffer. Deer facial (a-e) and leg (f-h) tissue and gland homogenate samples were adjusted to final protein concentrations of 1.5 μg/μL for the immunoassay. PrP^C^ signal was detected by anti-PrP SHA31 antibody.

Sample homogenates were not adjusted for protein concentration for the western blot analysis. Capillary electrophoresis immunoassay, using the anti-PrP SHA31 antibody, allowed for comparison of PrP^C^ protein expression between deer species and sex using total protein concentration-adjusted samples from clarified deer 10% (w/v) gland homogenates (Figure 5, Table 1, Supplementary Figures 3). Sex significantly influenced PrP^C^ expression in the forehead gland with expression higher in females than males. When the species was influential, white-tailed deer had higher levels of PrP^C^ expression. White-tailed deer expressed more PrP^C^ in the parotid, metatarsal, and interdigital glands than mule deer. Following significant interactions between species and sex, post-hoc tests indicated that white-tailed females had higher PrP^C^ expression in the parotid, nasal, and metatarsal glands than mule deer males, and white-tailed males expressed more PrP^C^ than mule deer males. Within species, white-tailed deer females expressed more PrP^C^ in the metatarsal glands than males, and mule deer males expressed PrP^C^ more than in females (Figure 5).

**Table 1.** Species and sex influence on PrP^C^ detection by anti-PrP SHA31 capillary gel electrophoresis assay with total protein concentration-standardized 10% (w/v) clarified gland homogenates

Exocrine	Sample Size						2-Way ANOVA P Values					
Tissue	Mule Deer			White-tailed Deer								
		Male	Female		Male	Female	Interaction		Species		Sex	
Vomeronasal	n = 10	n = 6	n = 4	n = 9	n = 4	n = 5	0.0241	*	0.1322	n.s.	0.5962	n.s.
Parotid	n = 11	n = 6	n = 5	n = 10	n = 6	n = 4	0.0250	*	0.0011	**	0.9608	n.s.
Forehead	n = 10	n = 5	n = 5	n = 11	n = 6	n = 5	0.6200	n.s.	0.3282	n.s.	0.0054	**
Preorbital	n = 11	n = 6	n = 5	n = 12	n = 6	n = 6	0.1997	n.s.	0.0564	n.s.	0.0517	n.s.
Nasal	n = 11	n = 6	n = 5	n = 12	n = 6	n = 6	0.0049	**	0.3594	n.s.	0.6823	n.s.
Tarsal	n = 11	n = 6	n = 5	n = 12	n = 6	n = 6	0.2779	n.s.	0.1540	n.s.	0.4555	n.s.
Metatarsal	n = 11	n = 5	n = 6	n = 12	n = 6	n = 6	0.0050	**	0.0106	*	0.3302	n.s.
Interdigital	n = 12	n = 6	n = 6	n = 11	n = 6	n = 5	0.2029	n.s.	0.0002	***	0.4598	n.s.

PrP^C^-associated chemiluminescent baseline-fit corrected peak areas were compared between species and sex.

2-Way ANOVA significance indicators: n.s. p ≥ 0.05, * p < 0.05, ** p < 0.01, *** p < 0.001

### Protein PrP^C^ concentration quantitation by ELISA:

**Figure 6.**
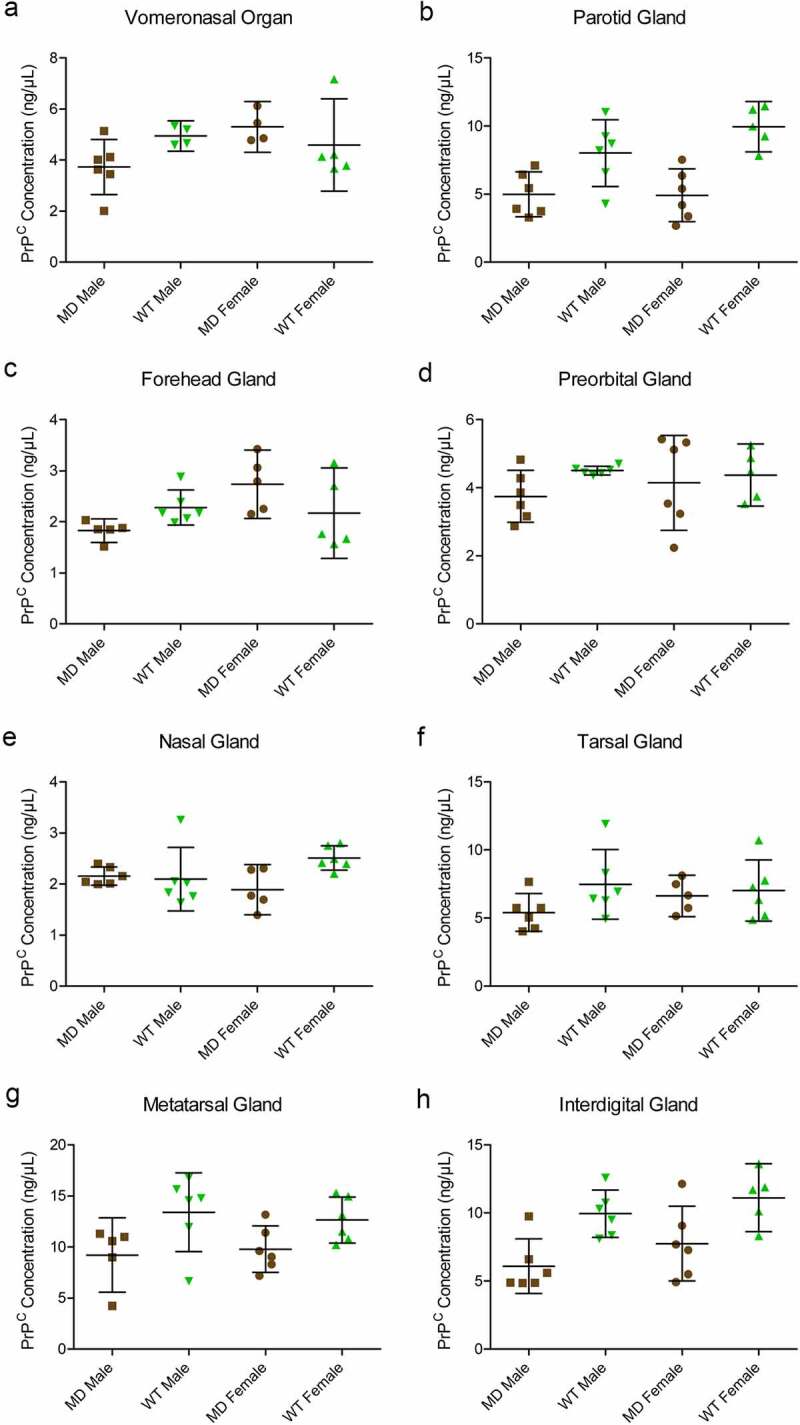
Quantified PrP^C^ protein concentration in deer gland homogenates. Total PrP^C^ concentrations of individuals, means, and 95% confidence intervals of clarified 10% (w/v) mule deer (MD) and white-tailed deer (WT) facial (a-e) and leg (f-h) gland homogenates prepared in RIPA buffer as determined by SHA31-N5 sandwich ELISA. Protein concentration was calculated using a full-length recombinant deer prion protein standard curve.

The SHA31-N5 sandwich ELISA combination used for this study is limited to the detection of full-length and C2 PrP^C^ fragments. Average PrP^C^ protein concentrations of homogenates (not adjusted for total protein concentration) ranged from 2.03 ng/μL in mule deer nasal glands to 13.02 ng/μL in white-tailed deer metatarsal glands (Figure 6, Table 2). White-tailed deer 1% whole-brain homogenate control PrP^C^ concentration averaged 4.78ng/μL between the 8 SHA31-N5 ELISAs. When the homogenates are adjusted for protein concentration, 10% (w/v) brain homogenate contains 3.7–23.5 fold more PrP^C^ than the clarified 10% (w/v) deer glands. Species but not sex influenced PrP^C^ concentration in tissues. White-tailed deer expressed more PrP^C^ than mule deer in the parotid, metatarsal, and interdigital glands than mule deer. Following significant interactions between species and sex, post-hoc tests indicated that white-tailed females expressed more PrP^C^ in the nasal gland than mule deer females, and that mule deer females expressed more PrP^C^ in the forehead gland than male mule deer (Figure 6(c,e)).

**Table 2.** Mean PrP^C^ concentrations and 95% confidence intervals of clarified 10% (w/v) gland homogenates as determined by SHA31-N5 sandwich ELISA

Exocrine	PrP^C^ Concentration (ng/µL)						2-Way ANOVA P Values		
Tissue	Mule Deer			White-tailed Deer					
		Male	Female		Male	Female	Interaction	Species	Sex
Vomeronasal	4.36 ± 0.84	3.73 ± 1.08	5.30 ± 1.00	4.75 ± 0.82	4.95 ± 0.59	4.49 ± 1.81	0.0579	0.5961	0.2159
	n = 10	n = 6	n = 4	n = 9	n = 4	n = 5	n.s.	n.s.	n.s.
Parotid	4.96 ± 1.04	4.99 ± 1.65	4.92 ± 1.94	8.89 ± 1.45	8.01 ± 2.44	9.95 ± 1.86	0.2116	<0.0001	0.2441
	n = 12	n = 6	n = 6	n = 11	n = 6	n = 5	n.s.	****	n.s.
Forehead	2.28 ± 0.44	1.83 ± 0.23	2.74 ± 0.67	2.23 ± 0.34	2.28 ± 0.34	2.17 ± 0.89	0.0261	0.7878	0.0731
	n = 10	n = 5	n = 5	n = 11	n = 6	n = 5	*	n.s.	n.s.
Preorbital	3.95 ± 0.66	3.75 ± 0.76	4.15 ± 1.40	4.45 ± 0.32	4.51 ± 0.13	4.37 ± 0.91	0.4621	0.1812	0.7143
	n = 12	n = 6	n = 6	n = 11	n = 6	n = 5	n.s.	n.s.	n.s.
Nasal	2.03 ± 0.21	2.16 ± 0.18	1.89 ± 0.49	2.30 ± 0.30	2.10 ± 0.62	2.51 ± 0.24	0.0469	0.0962	0.6493
	n = 11	n = 6	n = 5	n = 12	n = 6	n = 6	*	n.s.	n.s.
Tarsal	5.96 ± 0.92	5.41 ± 1.39	6.63 ± 1.52	7.25 ± 1.39	7.47 ± 2.55	7.02 ± 2.24	0.3013	0.1342	0.6316
	n = 11	n = 6	n = 5	n = 12	n = 6	n = 6	n.s.	n.s.	n.s.
Metatarsal	9.52 ± 1.63	9.21 ± 3.64	9.78 ± 2.28	13.02 ± 1.84	13.40 ± 3.86	12.64 ± 2.26	0.5771	0.0071	0.9359
	n = 11	n = 5	n = 6	n = 12	n = 6	n = 6	n.s.	**	n.s.
Interdigital	6.93 ± 1.50	6.09 ± 2.01	7.76 ± 2.75	10.48 ± 1.23	9.94 ± 1.75	11.12 ± 2.49	0.7841	0.0006	0.1187
	n = 12	n = 6	n = 6	n = 11	n = 6	n = 5	n.s.	***	n.s.

Tissue homogenates were not adjusted for total protein concentration.

2-Way ANOVA significance indicators: n.s. p ≥ 0.05, * p < 0.05, ** p < 0.01, *** p < 0.001, **** p < 0.0001

### Distribution of PrP^C^ in exocrine glands:

**Figure 7.**
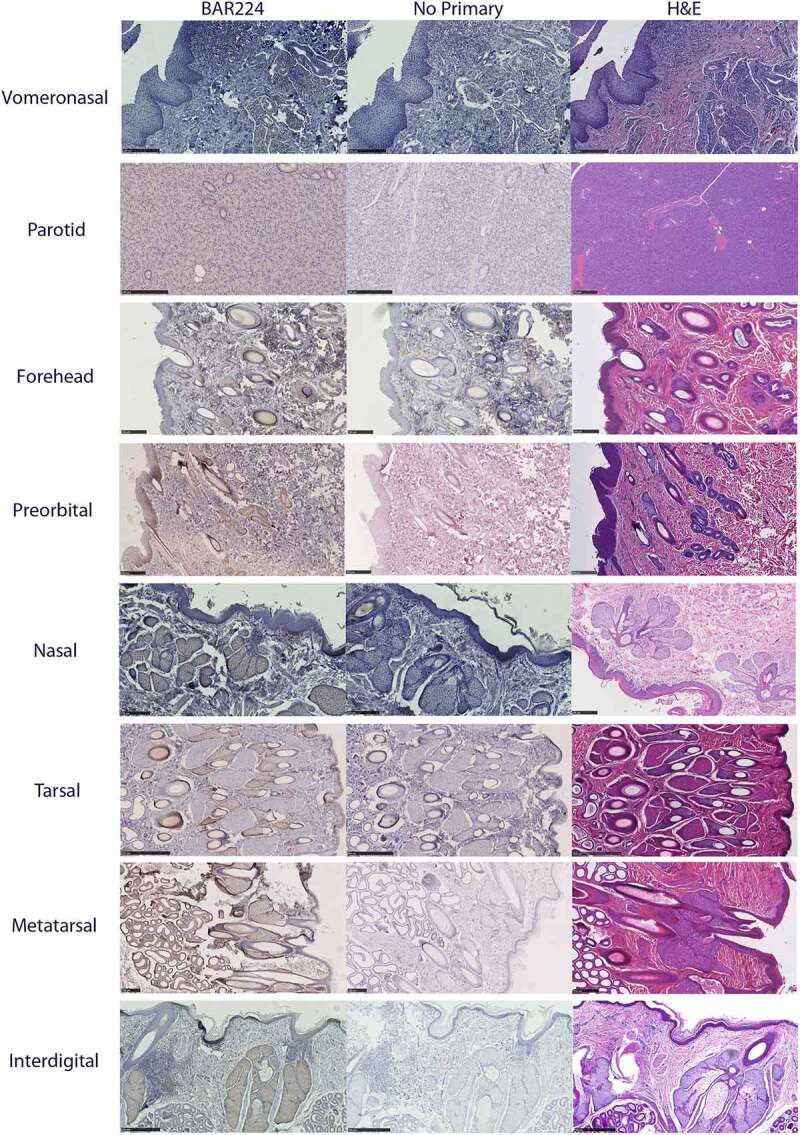
Tissue structure PrP^C^ distribution in mule deer and white-tailed deer exocrine glands. Anti-PrP BAR224 immunohistochemistry is contrasted with haematoxylin counterstain. BAR224 and no-primary antibody control immunohistochemistry slides of each gland were developed in the same batches. Scale bars for tarsal glands: 500 µm. All other scale bars: 250 µm.

Glandular PrP^C^ distribution was visualized by immunohistochemistry using the anti-PrP BAR224 monoclonal antibody. PrP^C^-associated immunohistochemistry intensity and glandular structures were confirmed by no-primary antibody negative controls and H&E staining (Figure 7). No differences were observed in the distribution of PrP^C^ within specific glandular structures between deer species or sex, although it must be noted that a smaller number of deer were selected for histological examination relative to the number used for the biochemical characterization methods.

**Figure 8.**
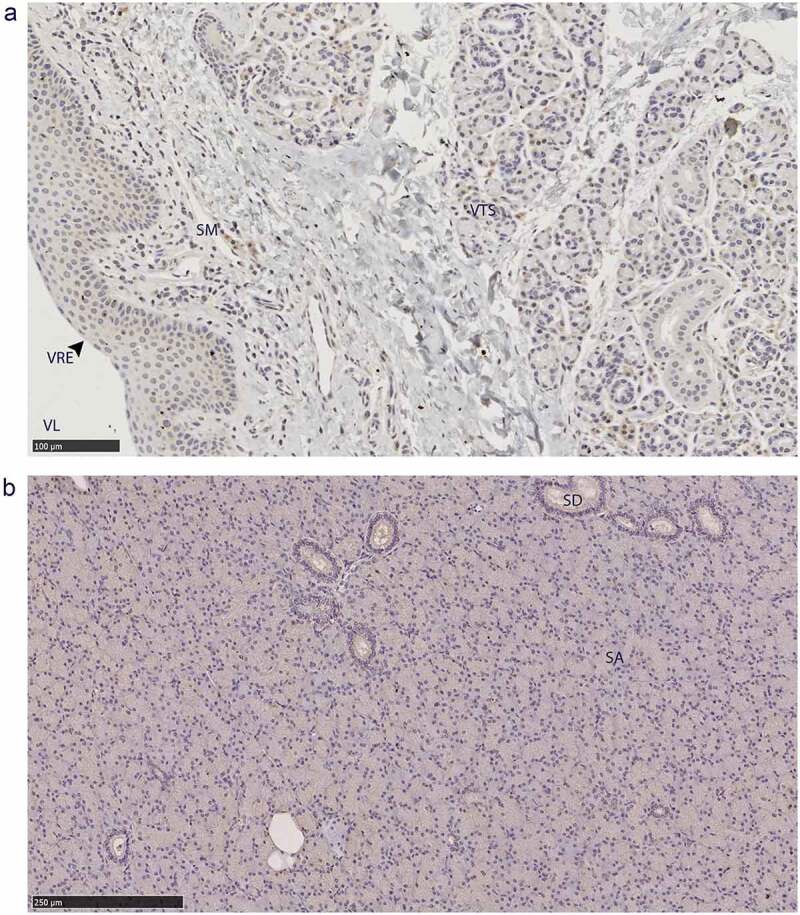
PrP^C^ distribution within non-integumentary facial glands. Immunohistochemical BAR224 detection of PrP^C^ (brown) with haematoxylin counterstaining. Female mule deer A) vomeronasal organ, and B) parotid gland. Structure abbreviations: SA, serous acini; SD, striated duct; SM, submucosa; VL, vomeronasal lumen; VRE, vomeronasal respiratory epithelium; VTS, vomeronasal tubular serous glands.

The non-integumentary vomeronasal organ presented with PrP^C^ in the tubular serous glands and the vomeronasal respiratory epithelium, immune cell infiltrates in the submucosa (Figure 8(a)). This study was limited to anterior sections of the vomeronasal organ. The sections we examined did not contain any vomeronasal sensory epithelium – consistent with anterior sections of Scandinavian moose vomeronasal organs [45]. Parotid gland PrP^C^ immunolabeling was disseminated throughout the glandular serous acini (Figure 8(b)).

**Figure 9.**
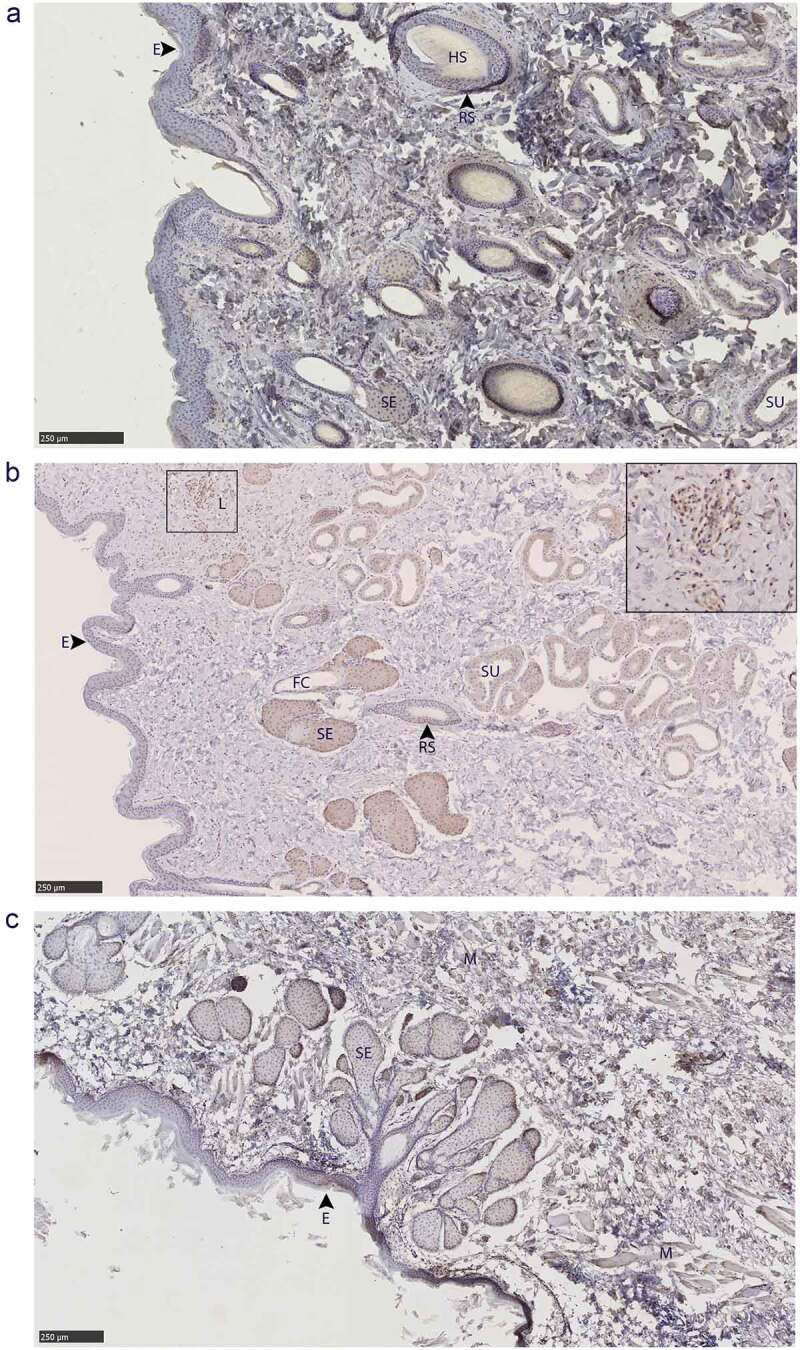
PrP^C^ distribution within facial integumentary glands. Immunohistochemical BAR224 detection of PrP^C^ (brown) with haematoxylin counterstaining. White-tailed male deer A) forehead gland, B) preorbital gland with magnified inset of infiltrating leukocytes, and mule deer female C) lateral vestibular nasal gland. Structure abbreviations: E, epidermis; FC, follicular canal; HS, hair shaft; L, leukocytic infiltrates; M, skeletal muscle; RS, follicular root sheath; SE, sebaceous glands; and SU, sudoriferous glands.

**Figure 10.**
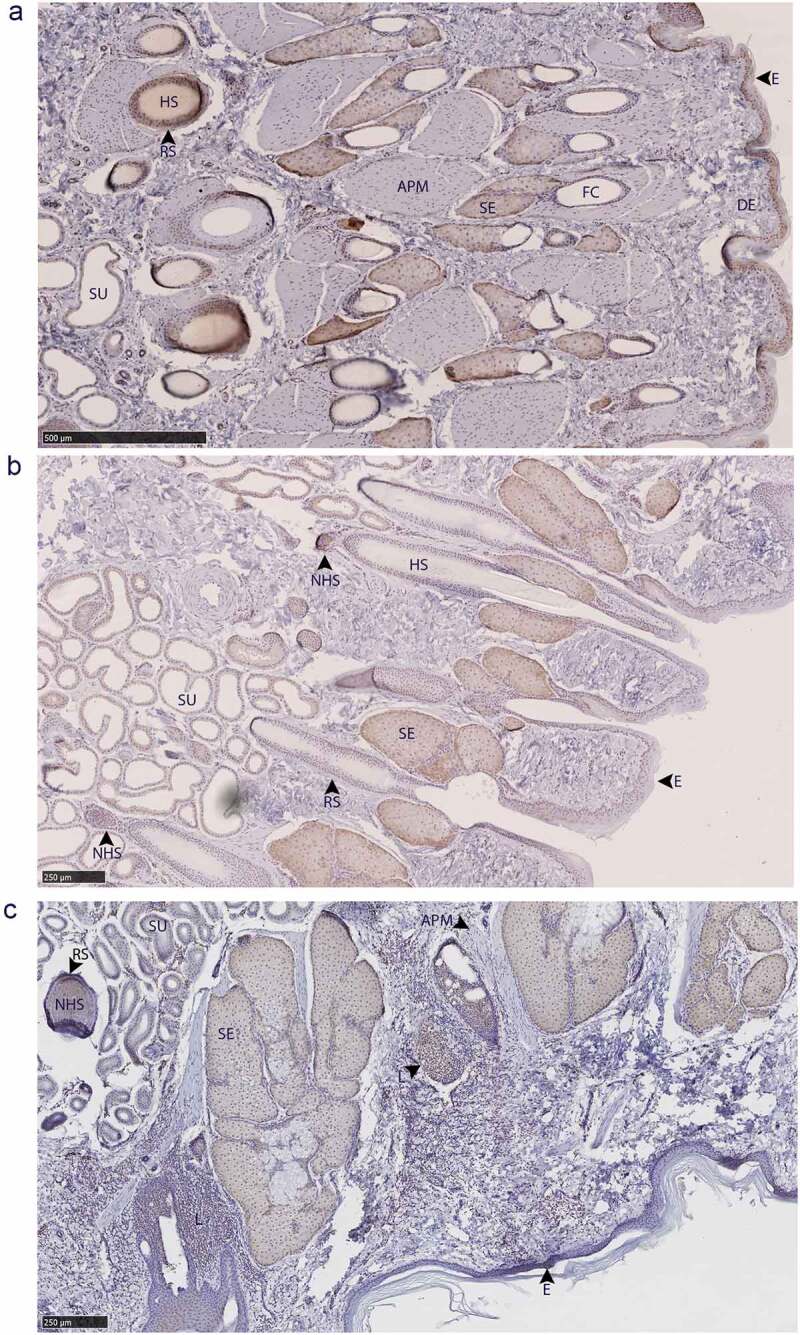
PrP^C^ distribution within leg integumentary glands. Immunohistochemical BAR224 detection of PrP^C^ (brown) with haematoxylin counterstaining. White-tailed male deer A) tarsal gland, B) metatarsal gland, and mule deer female C) interdigital gland. Structure abbreviations: APM, arrector pili muscle; DE, dermis elastic layer; E, epidermis; FC, follicular canal; HS, hair shaft; L, leukocytic infiltrates; NHS, nucleated region of the hair shaft; RS, follicular root sheath; SE, sebaceous glands; and SU, sudoriferous glands.

All six integumentary exocrine glands examined contained PrP^C^ in the holocrine sebaceous glandular cells, nearby epidermis, and hair root sheaths, and the nucleated regions of the hair follicles except for the dermal papilla (Figures 9,10). Sudoriferous glands were observed in all integumentary glands with the exception of the vestibular nasal glands. Contrary to Atkeson, *et al*., who reported the nasal glands of white-tailed deer and mule deer to be hairless epithelium [46], we observed hair follicles directly adjacent to the lateral vestibular nasal glands (Figure 7). Of a cautionary note for future surveys, DAB labelling can be mistaken for melanocytes among hair follicle matrix cells surrounding the hair bulb papilla and in the respiratory epithelium of vomeronasal organs.

Immunolabeling was observed in sudoriferous gland tubules of all integumentary glands except in the lateral vestibular nasal gland where these structures were absent. Non-integumentary nasal mucosa serous glands and skeletal muscle immunolabeled for PrP^C^ in sections of the vestibular nasal glands. Similarly, PrP^C^ immunolabeling was observed in the skeletal muscle of forehead gland sections. Sebaceous gland arrector pili smooth muscles were devoid of PrP^C^ immunolabeling. Collections of infiltrating leukocytes colocalized with PrP^C^ immunolabeling were observed in the preorbital and interdigital gland submucosa. The interdigital glands of mule deer contained notably abundant PrP^C^-expressing leukocytes between sudoriferous ducts, sebaceous glands, and in large leukocytic infiltrates (Figure 10(c)). Leukocytic infiltrates were present in the interdigital glands of white-tailed deer, but less conspicuous. The remarkably concentrated presence of immune cells in mule deer interdigital glands is consistent with prior cervid integumentary gland observations [23]. The interdigital gland sebaceous zone was thicker in white-tailed deer compared to mule deer – consistent with past observations [23]. Structurally, the interdigital glands of white-tailed deer have more developed sebaceous glands, but less developed sudoriferous glands than mule deer – consistent with observations by Quay [23,47].

Protozoal *Sarcocystis sp*. parasitic cysts were observed in the non-glandular skeletal muscle portions of the vestibular nasal, preorbital, and forehead glands of white-tailed deer and mule deer. The presence of the *Sarcocystis sp*. sarcocysts is expected in the context of CFB Wainwright observations in the 1970ʹs of the parasite in white-tailed deer and mule deer [48]. The parasite’s presence in specific organs is considered extraneous with respect to CWD or any gland-associated behaviours given that muscular invasion within cervids follows oral sporocyst ingestion. Sarcocysts observed were not associated with inflammation that could be expected to recruit PrP^CWD^-containing infiltrates.

## Discussion:

The presence and distribution of white-tailed deer and mule deer PrP^C^ within six integumentary facial and leg glands, and the non-integumentary vomeronasal organ and parotid gland were investigated to assess their potential for the disease-associated PrP^CWD^ isoform uptake or shedding. We sought to determine the presence and distribution of PrP^C^ within the tissues. Secondarily, we were interested if PrP^C^ abundance corresponds to the sex and species differences in CWD prevalence. Mule deer, and male cervids, have been consistently observed to be more prone to CWD infection [14–19].

PrP^C^ was detected by multiple methods in all eight tissues examined in this survey. Based on western blot, the clarified 10% (w/v) gland homogenates are estimated to possess approximately 10–25 fold less PrP^C^ than 10% (w/v) brain homogenates. Similarly, sandwich ELISA determined the gland homogenates to contain between 3.7 and 23.5 fold less PrP^C^ than brain homogenate – the difference in estimates being attributed to restricted PrP^C^ fragment detection by sandwich ELISA relative to western blot. Time between the animals being killed by hunters and sample collection may have contributed to individual variation of PrP^C^ detection via protein degradations. Protein degradation being a factor in PrP^C^ detection was mitigated by cold ambient temperature during the hunt and by the preferential selection of samples originating from deer with shorter periods of time between death and sampling. The deer examined in this study were sampled exclusively near the end of the breeding season. The possibility that PrP^C^ protein expression and gland activity varies cyclically throughout the year cannot be excluded.

Contrary to CWD wild population prevalence patterns, we observed that glandular PrP^C^ expression was significantly higher in females or in white-tailed deer – regardless of whether homogenate samples were adjusted for total protein content. Western blot analysis determined that female deer of both species expressed more PrP^C^ in the forehead and preorbital glands than male deer (Figure 3(a-d)). Capillary electrophoresis immunoassay of protein concentration-adjusted samples confirmed the western blot analysis of the females having more PrP^C^ expression in the forehead glands, and also indicated that white-tailed deer express more PrP^C^ than mule deer in the interdigital glands (Table 1). ELISA quantification of unadjusted gland homogenates similarly indicated that white-tailed deer express more PrP^C^ than mule deer in the parotid, metatarsal, and interdigital glands (Table 2). Discrepancies in tissues identified to have significant PrP^C^ expression differences between the capillary electrophoresis and ELISA methods can be explained by a lack of total protein adjustment in ELISA and fewer PrP^C^ molecular species being detected by the sandwich ELISA relative to the single antibody-based detection of western blot or capillary electrophoresis immunoassays.

The lack of correlation between deer gland PrP^C^ expression and known CWD sex and species prevalence patterns are not the only variables to be considered. Our determination of PrP^C^ concentrations do not take into consideration glandular size and secretion differences between season, species, and sex. For example, western blot and capillary electrophoresis immunoassay determined that females express more PrP^C^ than males; however, male deer have thicker forehead glands [22]. Likewise, white-tailed deer were identified as having significantly more PrP^C^ expression in the metatarsal glands by ELISA and capillary electrophoresis immunoassay, but this may be counterbalanced by mule deer having larger metatarsal glands [21,44]. Capillary electrophoresis and ELISA determined that white-tailed deer express more PrP^C^ than mule deer – correlating with our and historical observations of white-tailed deer interdigital glands secreting more actively [21].

Surveys of cervid PrP^C^ expression and distribution in non-neuronal tissues is limited, but has been examined in the alimentary tract and associated lymph nodes in white-tailed deer [5]. Davenport *et al*. determined that all examined tissues variably expressed PrP^C^ and accumulated PrP^CWD^ prion seeding activity in symptomatic animals. The authors also determined that higher relative PrP^C^ expression between tissues did not correlate well with early preclinical PrP^CWD^ prion seeding activity. Relative sex and species expression differences between the six integumentary glands and two non-integumentary tissues were generally low in magnitude; PrP^C^ was readily detectable in all deer tested and ELISA quantification of PrP^C^ concentrations determined that all differences were less than two-fold (Table 2). Although yet to be investigated, all tissues examined expressed PrP^C^ at levels that could feasibly replicate PrP^CWD^. The PrP^C^ expression differences of the tissues investigated may not be sufficient in magnitude to influence horizontal CWD transmission and broad disease prevalence patterns.

Immunohistochemistry of the integumentary gland sections identified PrP^C^ localized in the integumentary glandular cells, hair follicles, epidermis near glandular ducts, and local immune cells. Resident immune cells – especially the large clusters observed in the interdigital glands – and *in situ* nerve endings are envisioned to be accommodating to prion conversion and trafficking. Many of the structures identified in the exocrine gland sections that labelled for PrP^C^ are composed of actively replicating cells that would be anticipated to have a low propensity for PrP^CWD^ replication based on cell division rate [49]. Although the glandular cells and hair follicles are suspected to be poorly tropismatic for PrP^CWD^, these integumentary structures are innervated in cervids [23,50,51] which could permit direct nervous system access for PrP^CWD^ uptake or shedding. Parotid glands of orally infected deer at clinical stage of disease were observed to have PrP^CWD^ in parasympathetic nerves and the interstitial space of the gland rather than the acini or glandular ducts [52]. Supportive of infectious prion replication in glandular cells is the observation of PrP^Sc^ of scrapie-infected sheep in the parotid gland acinar cells, ductal cells, and interstitia – depending on the antibody used [53]. PrP^CWD^ labelling by 6H4 in the deer study is analogous to PrP^SC^ labelling by L42 in scrapie-infected sheep work. The anti-prion protein 6H4 monoclonal antibody has an epitope that encapsulates the epitope of L42. Both antibodies identified infectious prions in the interstitium of the parotid gland. Alternative anti-prion antibody use in deer may identify PrP^CWD^ in the acinar cells and glandular ducts of the parotid gland (and other glands) as observed in scrapie.

The integumentary glands examined expressed similar levels of PrP^C^ to the parotid gland, which is known to accumulate PrP^CWD^ [6,31,34,52]. PrP^Sc^ has been observed in the vomeronasal organ sensory epithelium, but not the respiratory epithelium of prion infected hamsters [54]. To be considered for future studies is the lack of sensory epithelium in the anterior portions of the vomeronasal organ. Although PrP^C^ expression was lower than brain homogenate, the eight tissues examined expressed PrP^C^ at levels that could feasibly replicate PrP^CWD^. The PrP^C^ expression in the glands examined (of both species and sex), therefore, offer CWD transmission potential that has yet to be investigated.

Species and sex differences in cervid behaviours including grouping and movement patterns, sparring, courtship behaviour, scent-marking, and other advertisement activities have been previously proposed to explain the disease prevalence differences of wild animal populations [16,37–39]; however, uniting cervid behaviours, physiology, and CWD transmission is currently lacking. Our identification of PrP^C^ presence in the vomeronasal organ and integumentary scent glands associated with mule deer and white-tailed deer behaviours provides a biochemical foundation for understanding these tissues if they are involved in CWD transmission. Investigations into the presence of PrP^CWD^ in the described tissues will provide further insights into possible mechanisms of CWD transmission that drive asymmetrical disease prevalence. Investigations into PrP^CWD^ within cervid exocrine glands could reveal valuable insights into the mechanisms of CWD transmission that drive asymmetrical disease prevalence.

## Supplementary Material

Supplemental Material
