# Supplementary figures and images for: Cellular prion protein distribution in the vomeronasal organ, parotid, and scent glands of white-tailed deer and mule deer
Source: Prion. 2022 May 29;16(1):40–57. doi: 10.1080/19336896.2022.2079888 (PMC9154781; doi:10.1080/19336896.2022.2079888)

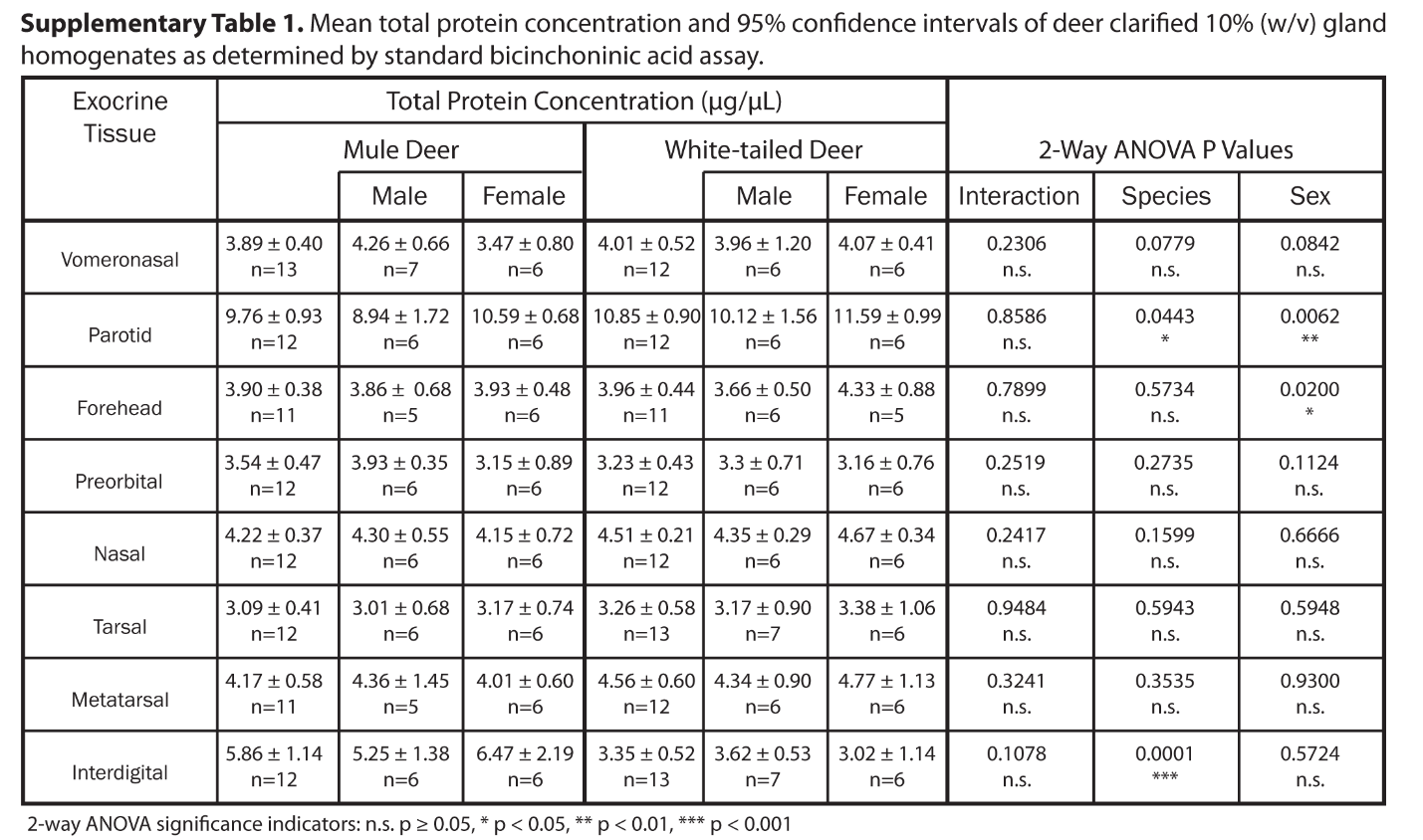


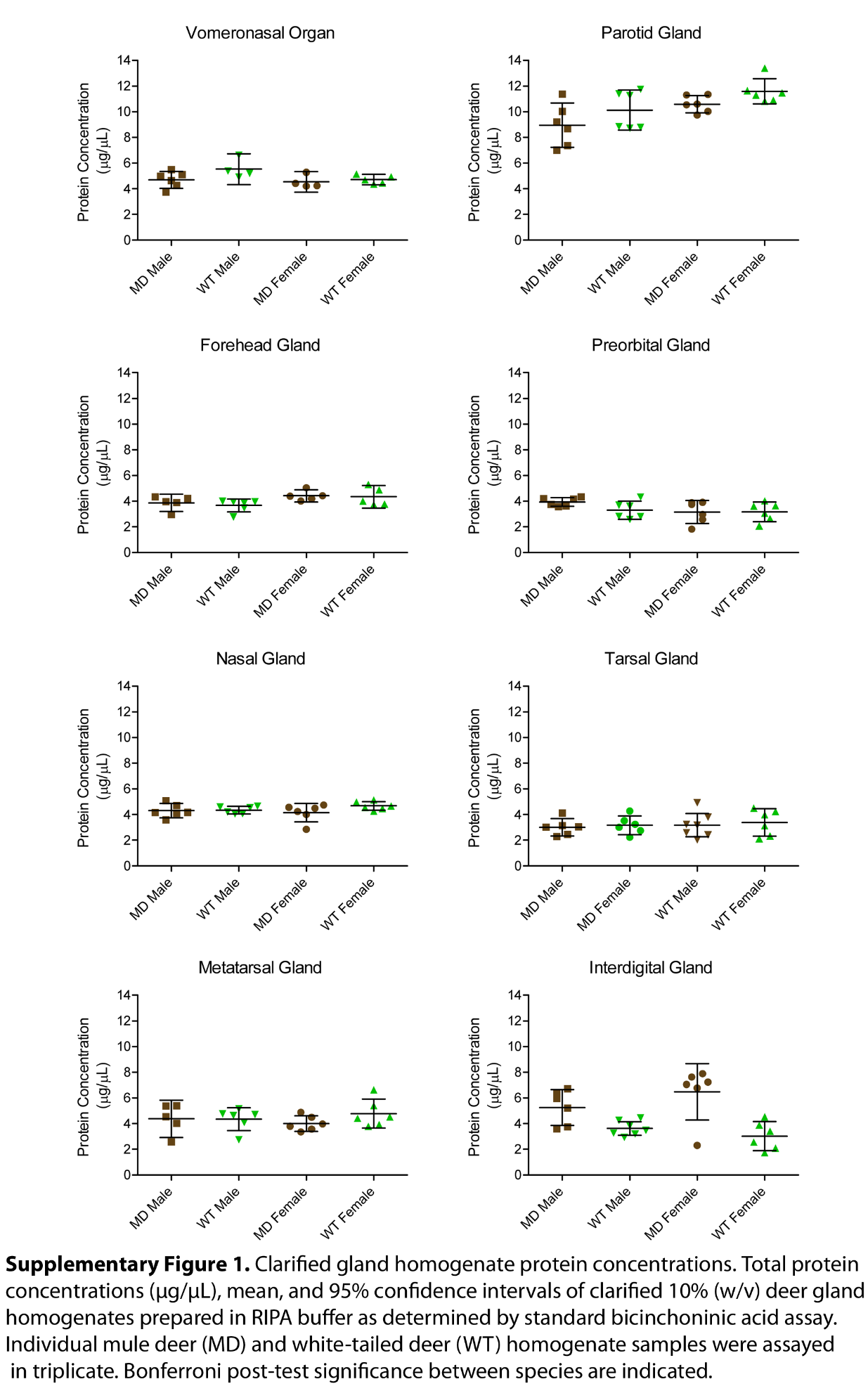


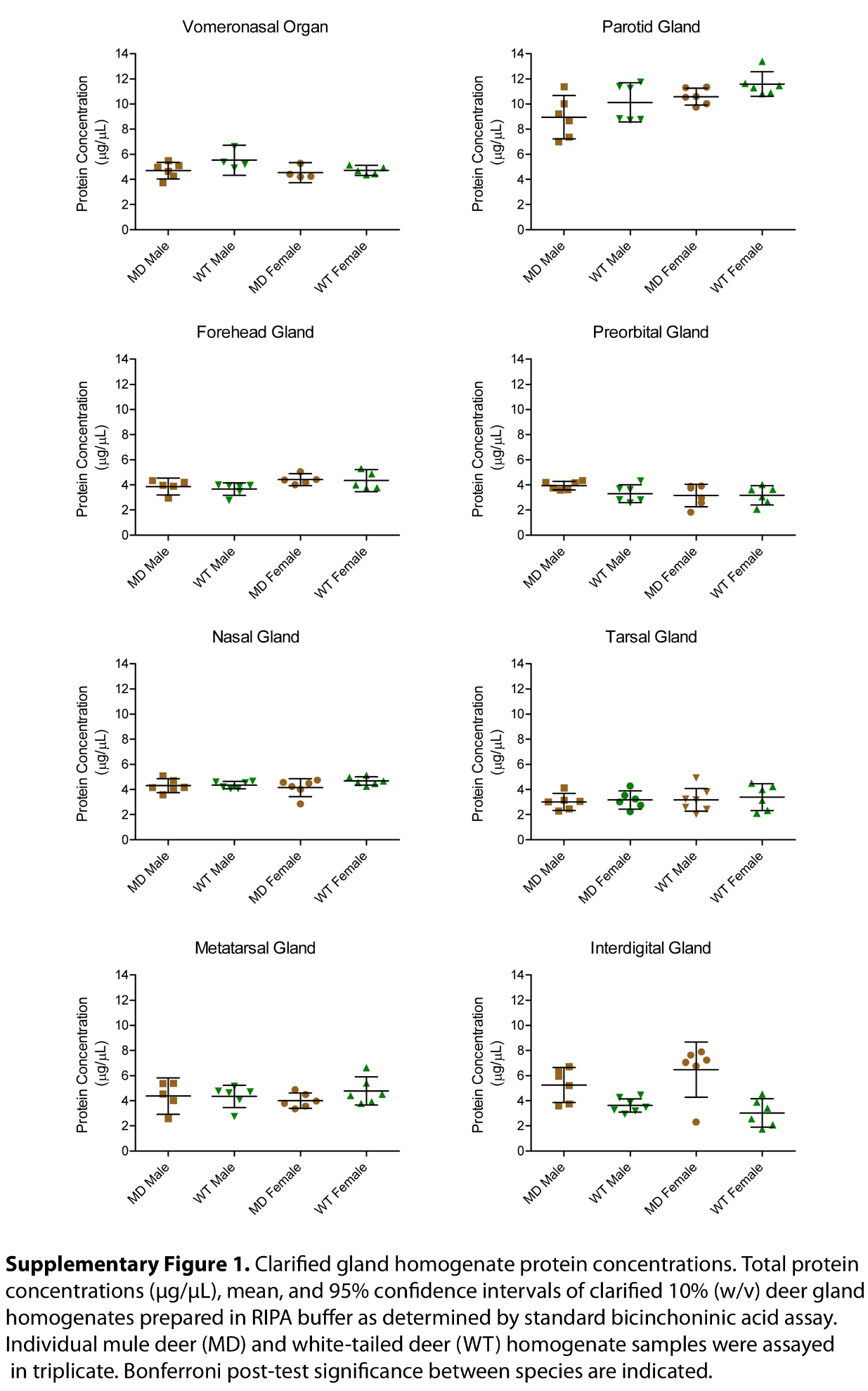


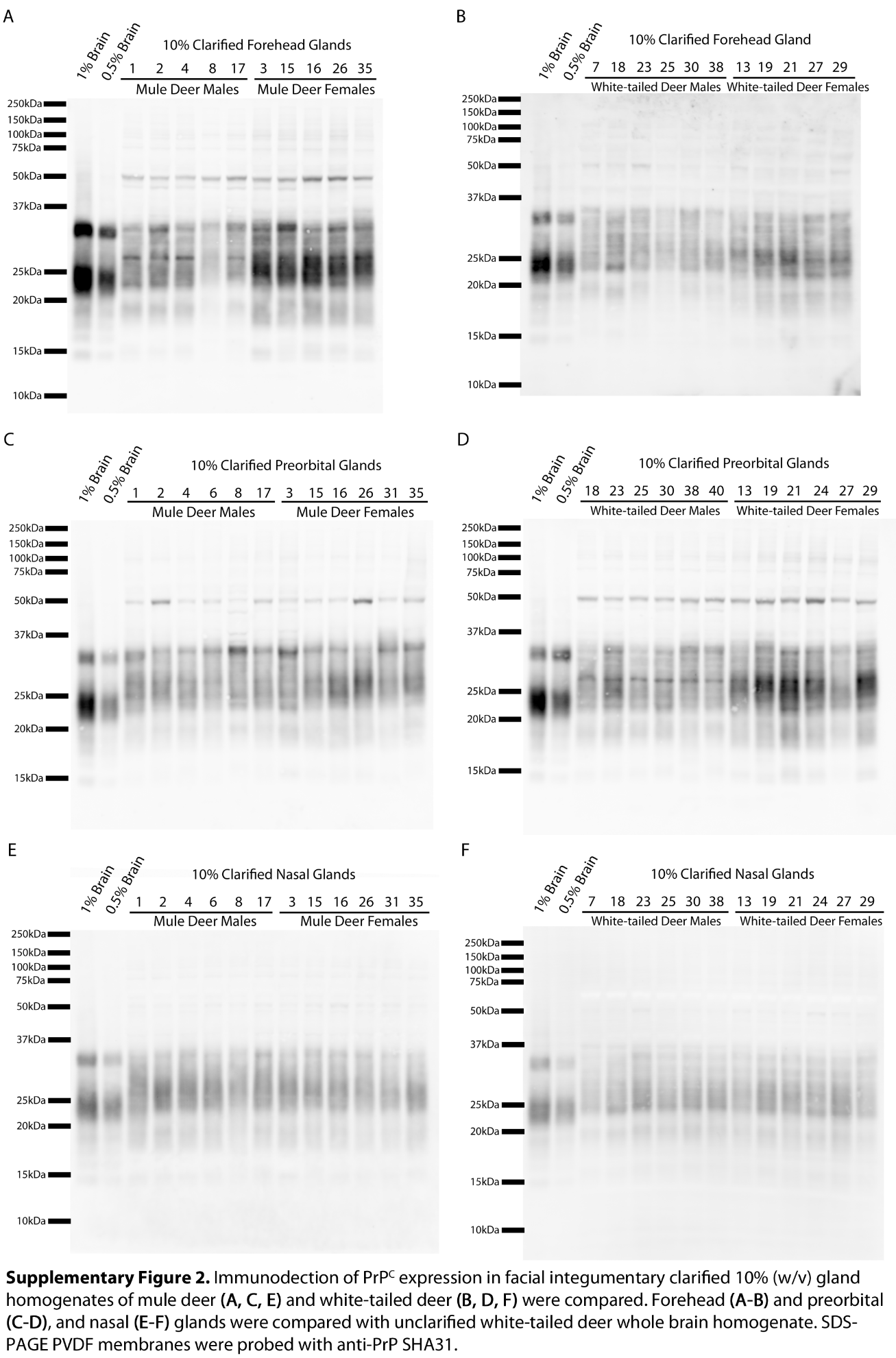


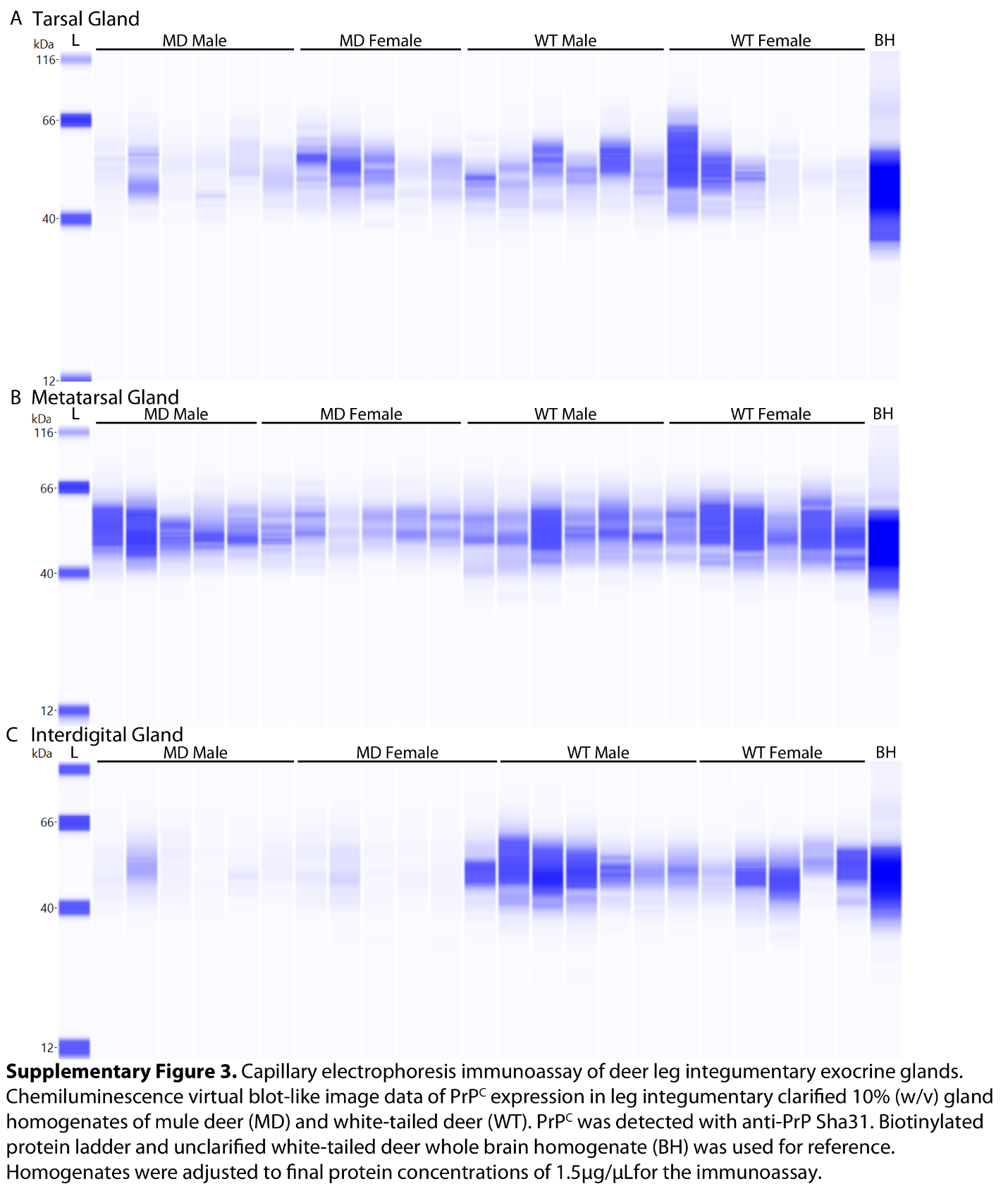

Supplement: Supplemental Material [file KPRN_A_2079888_SM2064.zip › Supplementary Materials.docx]
